# Perceived general, mental, and physical health of Latinos in the United States following adoption of immigrant-inclusive state-level driver’s license policies: a time-series analysis

**DOI:** 10.1186/s12889-022-14022-x

**Published:** 2022-08-24

**Authors:** Cristian Escalera, Paula D. Strassle, Stephanie M. Quintero, Ana I. Maldonado, Diana Withrow, Alia Alhomsi, Jackie Bonilla, Veronica Santana-Ufret, Anna María Nápoles

**Affiliations:** Division of Intramural Research, National Institute On Minority Health and Health Disparities, National Institutes of Health, Building 3, Room 5E08, 3 Center Drive, Bethesda, MD 20892 USA

**Keywords:** Latino health, State policies, Immigrant health, Driver’s license, Health disparities

## Abstract

**Background:**

In the United States (U.S.), several states have laws that allow individuals to obtain driver’s licenses regardless of their immigration status. Possession of a driver’s license can improve an individual’s access to social programs, healthcare services, and employment opportunities, which could lead to improvements in perceived mental and physical health among Latinos living in the U.S.

**Methods:**

Using Behavioral Risk Factor Surveillance System data (2011–2019) for Latinos living in the U.S. overall (immigration status was not available), we compared the average number of self-reported perceived poor mental and physical health days/month, and general health status (single-item measures) before (January 2011-June 2013) and after implementation (July 2015-December 2019) of immigrant-inclusive license policies using interrupted time-series analyses and segmented linear regression, and a control group of states in which such policies were not implemented. We also compared the average number of adults reporting any perceived poor mental or physical health days (≥ 1 day/month) using a similar approach.

**Results:**

One hundred twenty-three thousand eight hundred seven Latino adults were included; 66,805 lived in states that adopted immigrant-inclusive license policies. After implementation, average number of perceived poor physical health days significantly decreased from 4.30 to 3.80 days/month (immediate change = -0.64, 95% CI = -1.10 to -0.19). The proportion reporting ≥ 1 perceived poor physical and mental health day significantly decreased from 41 to 34% (OR = 0.89, 95% CI = 0.80–1.00) and from 40 to 33% (OR = 0.84, 95% CI = 0.74–0.94), respectively.

**Conclusions:**

Among all Latinos living in the U.S., immigrant-inclusive license policies were associated with fewer perceived poor physical health days per month and fewer adults experiencing poor physical and mental health. Because anti-immigrant policies can harm Latino communities regardless of immigration status and further widen health inequities, implementing state policies that do not restrict access to driver licenses based on immigrant status documentation could help address upstream drivers of such inequities.

## Background

In 2019, it was estimated that over 60 million Latino individuals lived in the United States (U.S.), roughly 19% of the total U.S. population [1]. Twelve states had a population of one million or more Latino residents in 2019; California, Texas, Florida, New York and Arizona were the top five states, with four of these bordering Mexico [2]. Almost 22% (13 million) of Latino persons in the country are not U.S. citizens [3], however estimating the number of undocumented (do not possess a valid visa or other immigrant documentation) immigrants is difficult. The Department of Homeland Security estimates that roughly 13% (under 9 million) of Latinos in the U.S. are undocumented [4].

In the U.S., state-level immigration policies increasingly affect the lives of Latino immigrants [5, 6]. State-level policies can either increase or constrain immigrants’ access to services and benefits. Additionally, state immigration policies shape the immigrant experience of settlement and incorporation and reflect the state’s position towards immigrants [6, 7]. One example of immigrant-inclusive policies are state laws that allow for the issuing of driver’s licenses regardless of legal immigration status, henceforth referred to as immigrant-inclusive policies. As of December 2021, 16 U.S. states and the District of Columbia have such policies [8, 9].

**Fig. 1:**
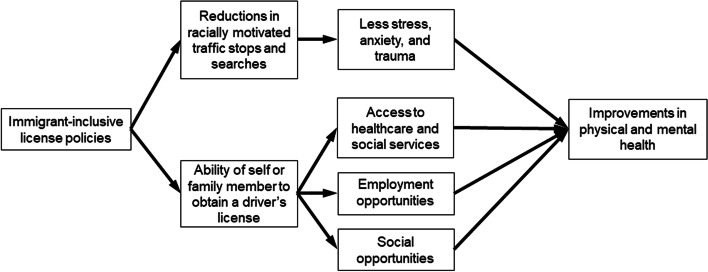
Conceptual framework for how states enacting immigrant-inclusive license policies could positively impact the physical and mental health of Latinos in the United States

Allowing immigrants, regardless of citizenship or legal documentation status, to obtain driver’s licenses has the potential to impact the general, physical, and mental health of Latinos (Fig. 1). For undocumented immigrants, possessing a driver’s license can improve access to healthcare and social services, and social, recreational and employment opportunities [7], and conversely, requiring a form of identification limits their ability to access public services [10]. Additionally, fears of deportation and detention that would be magnified when driving without a license, and associated elevated chronic stress could negatively impact general, physical, and mental health [11].

Anti-immigrant policies in the U.S. affect the health and well-being of both U.S.-born and immigrant Latinos, and documented and undocumented noncitizens, due to spillover effects and because citizenship status cannot be visually ascertained[11]. Because half of all undocumented Latinos live in mixed-status families (e.g., parents may be undocumented while children have legal status in the U.S.) [12], some of the health benefits associated with undocumented immigrants’ ability to obtain a driver’s license could extend to Latinos who are U.S. born, naturalized, and/or legal residents. Furthermore, anti-immigrant policies that reflect or engender anti-immigrant attitudes and racial profiling have been shown to generate equal levels of psychological distress among U.S.-born and immigrant Latinos [13]. Finally, legal enforcement of driver’s license policies, e.g., traffic stops by police, affect all Latinos, including legal residents and U.S. citizens. One study employing complex standardization and analyses of over 100 million traffic stops found that police require less suspicion during traffic stops to search Black and Hispanic drivers than White drivers, suggesting persistent racial bias [14]. In fact, racial profiling is considered a public health and health disparities issue in the U.S. because it can indirectly and directly cause adverse health consequences through stress, trauma and anxiety [15].

Despite the potential economic and health benefits of having access to a driver’s license, the impact of enacting immigrant-inclusive license policies on Latino health in the U.S. is currently unknown. Thus, the aim of the current study was to measure the impact of enacting immigrant-inclusive license policies on the physical, mental, and general health of all Latinos (undocumented, legal residents, and U.S. citizens) living in the U.S. We hypothesized that these policy changes would be associated with better physical, mental, and general health among Latinos in the U.S. Because significant expansion of health insurance access occurred in some U.S. states at the same time as the enactment of immigrant-inclusive driver’s license policies (2013–2015), we only included states in this study where this health insurance expansion occurred. A major part of the Affordable Care Act (ACA), signed into law by President Obama in 2010 and implemented in 2014, was to expand eligibility criteria for Medicaid, a federal and state joint program that provides health insurance coverage for low-income citizens and documented immigrants (undocumented were not eligible), which disproportionately included Black and Latino individuals [16]. However, states were allowed to opt out of Medicaid expansion. Thus, we only included states that were similar in terms of Medicaid expansion but differed on adoption of immigrant-inclusive policies.

## Methods

### Data source and study population

We utilized data from the 2011–2019 Behavioral Risk Factor Surveillance System (BRFSS) survey, an ongoing, state-based, random-digit-dialed telephone survey of non-institutionalized U.S. adults (≥ 18 years old). BRFSS collects state data about health-related risk behaviors, chronic health conditions, and use of preventive services. The questionnaire consists of core questions asked in all 50 states, the District of Columbia, and U.S. territories. Data are weighted to reflect the age, sex, and racial/ethnic distribution of the state’s estimated population during each survey year. Data prior to 2011 was not included due to changes in weighting methodology and the addition of the cell phone sampling frame that occurred that year [17]. All participants that self-identified as being Hispanic or Latino ethnicity were included. Due to data limitations, we were unable to distinguish between Latinos who are undocumented, legal residents, and U.S. citizens.

### Measuring perceived physical and mental health among Latinos

Perceived physical and mental health were captured using two items which asked, “for how many days during the past 30 days was your physical (mental) health not good?” Perceived general health status was assessed using the question “Would you say that in general your health is?” Response options included excellent (1), very good (2), good (3), fair (4), and poor (5). Scoring for this question was reversed for analyses, so that higher scores indicated better perceived general health.

Other sociodemographic characteristics included state of residence, age, gender, marital status, education, employment status, annual household income, interview language, health insurance status, inability to seek care because of cost, and amount of time since last routine physical exam checkup.

### Identifying states that have immigrant-inclusive license policies between 2011 and 2019

Information on each state’s immigrant driver’s license policies were captured using reports from the National Conference of State Legislatures [8] and National Immigration Law Center [9]. Legislation was verified with corresponding state bills. States that have enacted immigrant-inclusive driver’s license polices (defined as state legislature that issues a license if an applicant provides certain documentation, such as foreign birth certificate, foreign passport, or consular card and evidence of current residency in the state) that allow undocumented immigrants to obtain licenses include: California, Colorado, Connecticut, Delaware, District of Columbia, Hawaii, Illinois, Maryland, New Mexico, Nevada, Oregon, Utah, Vermont, Virginia, and Washington. For our analyses, we excluded Latino participants living in Hawaii, New Mexico, Utah, and Washington because they had enacted their immigrant-inclusive license policies prior to 2011. Latino participants from Delaware were excluded due to small sample size (unweighted n = 2,072).

Because all license-expansion policies for the included intervention states were implemented between November 2013 and January 2015, time was stratified into three periods: pre-implementation (January 2011 – June 2013), during implementation (July 2013 – June 2015), and post-implementation (July 2015 – December 2019).

In order to remove the potential bias caused by the ACA and Medicaid expansion, we restricted our analysis to include only Latino participants living in states which opted into the expansion in 2014. This meant dropping Oregon and Virginia from the intervention state list, leaving our final group of included intervention states as: California, Colorado, Connecticut, Illinois, Maryland, Nevada, Vermont, and the District of Columbia (66,805 Latino adults).

We similarly restricted our control states (i.e., states that did not enact (and had not enacted previously) immigrant-inclusive license policies between 2011 and 2019) to those that participated in Medicaid expansion in 2014 as part of the ACA. Our final list of control states included: Arizona, Arkansas, Iowa, Kentucky, Massachusetts, Michigan, New Hampshire, New Jersey, New York, North Dakota, Ohio, Rhode Island, and West Virginia (57,002 Latino adults). Of note, New Jersey and New York enacted immigrant-inclusive license policies after the end of the study period (New Jersey: effective January 2021; New York: effective December 2019).

### Statistical analyses

Average number of perceived poor physical health days per month, perceived poor mental health days per month, and average perceived general health score were estimated at three-month intervals (i.e., quarterly or four data points per year) between 2011 and 2019 using data from the BRFSS survey. Descriptive statistics were used to compare BRFSS participant characteristics across these three time periods, stratified by intervention and control group status.

In order to assess the immediate and gradual effects of enacting statewide immigrant-inclusive license policies on Latino health, we conducted an interrupted time-series analysis; this quasi-experimental approach is commonly used to assess well-defined population-level changes (e.g., new laws or policies) when randomization is not possible [18, 19] Using segmented linear regression, we estimated rates and linear trends before and after immigrant-inclusive policies were implemented in the intervention states [19]. We compared both the change in slope (gradual change) and intercept (immediate change) during the post-implementation period (July 2015 – December 2019) to pre-implementation time (January 2011 – June 2013). A similar analysis comparing these two time periods was performed in the control states; if similar changes were observed among states that did not enact license-expansion policies it would suggest that differences were due to other secular policies or trends that impacted Latino health.

Based on our hypotheses, we expected the average number of perceived poor physical and mental health days per month to decrease and perceived general health to improve in the post-intervention period in states where license policies were expanded. We also expected that perceived physical and mental health would remain relatively consistent among states that did not implement immigrant-inclusive license policies.

We also performed two sensitivity analyses. First, we dichotomized our outcomes and modeled the proportion of adults reporting having any (≥ 1 versus none) perceived poor physical or poor mental health days per month, as well as the proportion of those with perceived poor general health (poor/fair versus good/very good/excellent). For these dichotomized analyses, logistic regression was used. Second, we restricted the sample to Latino participants who experienced at least 1 (i.e., any) poor physical or mental health days, treating the number of perceived poor health days as continuous. The hypothesis for these analyses was that license expansion may not necessarily reduce the number of adults (objective of first sensitivity analysis) with perceived poor health, but that among adults who reported *any* poor health days, living in states with immigrant inclusive driver’s license policies would be associated with *fewer* perceived poor physical and mental health days per month (objective of second sensitivity analysis).

Descriptive statistics were estimated using SAS version 9.4 (SAS Inc., Cary, NC) and segmented linear regression was performed using SUDAAN release 11.0.3 (Research Triangle Institute International, Research Triangle Park, North Carolina). All analyses accounted for the complex survey design of BRFSS and were weighted to obtain national estimates. Variances in the regression models were computed using the Taylor Linearization Method, assuming a with-replacement design, in order to account for the complex survey weights.

## Results

Overall, there were 123,807 Latino participants included in the analysis (intervention states: n = 66,805; control states: *n* = 57,002). A breakdown of participant demographics is reported in Table 1, stratified by status (intervention vs. control state) and time period (pre-, during, and post-implementation). Overall, demographics remained relatively consistent across time among participants living in both the intervention and control states. Participants from intervention states were slightly less likely to have a higher education, taken the BRFSS survey in English, and had a routine physical exam checkup in the past year.

**Table 1:** Demographics of Latino adults living in states that did and did not introduce immigrant-inclusive license policies between 2013–2015, stratified by study time period, weighted to be nationally representative, BRFSS 2011–2019

	Enacted immigrant-inclusive license policies^a^			Did not enact inclusive immigrant-inclusive policies^b^		
	Pre- Implementation	Implementation Period	Post-Implementation	Pre-Implementation	Implementation Period	Post-Implementation
**Age group, n (%)**						
18 to 24	1800 (17)	1578 (17)	4368 (16)	1782 (18)	1289 (17)	3417 (17)
25 to 34	3514 (26)	2664 (26)	7249 (25)	3265 (25)	2261 (25)	5648 (24)
35 to 44	4114 (22)	2944 (22)	7787 (21)	3488 (22)	2433 (21)	5807 (22)
45 to 54	3371 (16)	2593 (17)	6850 (17)	3378 (16)	2392 (16)	5084 (15)
55 to 64	2588 (11)	1827 (11)	5244 (12)	2477 (11)	1901 (12)	4173 (12)
65 or older	2418 (7)	1671 (8)	4225 (9)	2470 (8)	1898 (9)	3839 (10)
**Male, n (%)**	7294 (51)	5926 (50)	16,976 (50)	6660 (50)	4936 (49)	12,585 (49)
**Marital status, n (%)**						
Married/member of couple	10,274 (56)	7278 (56)	19,393 (56)	8120 (50)	5953 (49)	13,809 (50)
Divorced/separated	2793 (12)	2092 (12)	5618 (12)	3463 (14)	2442 (17)	5043 (15)
Widowed	1082 (3)	711 (3)	1615 (3)	1135 (3)	759 (4)	1491 (4)
Never married	3580 (29)	3096 (28)	8924 (28)	3964 (33)	2889 (31)	7410 (31)
**Highest education, n (%)**						
Less than high school	3122 (23)	2219 (23)	6432 (24)	2573 (18)	1812 (19)	3695 (18)
Some high school	2331 (19)	1734 (19)	4579 (17)	2025 (18)	1370 (17)	2862 (16)
High school graduate	4838 (26)	3669 (26)	9675 (26)	4975 (28)	3341 (27)	8028 (28)
Some college/technical school	4128 (22)	2895 (24)	7485 (23)	3852 (24)	2895 (24)	6912 (24)
College graduate	3170 (9)	2561 (9)	7415 (10)	3256 (13)	2602 (13)	6311 (14)
**Employment status, n (%)**						
Employed for wages	8170 (49)	6586 (51)	18,152 (52)	7807 (50)	5640 (49)	13,677 (51)
Self-employed	1272 (8)	1071 (8)	3420 (10)	1103 (8)	918 (9)	2415 (10)
Out of work, < 1 year	910 (6)	583 (5)	1428 (4)	861 (6)	475 (5)	1110 (4)
Out of work, ≥ 1 year	976 (6)	518 (5)	1066 (3)	929 (6)	506 (4)	972 (4)
Homemaker	2280 (12)	1448 (13)	3812 (12)	1449 (9)	1105 (10)	2348 (9)
Student	726 (7)	555 (7)	1623 (6)	734 (6)	534 (7)	1365 (7)
Retired	1952 (6)	1318 (6)	3313 (7)	1855 (7)	1462 (8)	3084 (8)
Unable to work	1257 (5)	935 (6)	2437 (6)	1892 (8)	1300 (9)	2554 (8)
**Annual household income, n (%)**						
< $15,000	3983 (27)	2590 (27)	5659 (22)	3373 (23)	2228 (23)	4062 (18)
$15,000 – $24,999	3929 (25)	2829 (25)	7072 (23)	4098 (30)	2867 (30)	6117 (29)
$25,000 – $34,999	2054 (14)	1407 (12)	3927 (14)	1862 (13)	1240 (13)	2805 (12)
$35,000 – $49,999	1922 (12)	1378 (12)	3811 (13)	1599 (12)	1133 (11)	2840 (12)
≥ $50,000	4084 (22)	3225 (24)	9272 (28)	3166 (22)	2527 (23)	6733 (28)
**Interview language, n (%)**						
English	11,927 (59)	8730 (60)	22,111 (56)	10,674 (63)	7888 (62)	19,361 (65)
Spanish/Other	5876 (41)	4387 (40)	13,610 (44)	6028 (37)	4262 (38)	8602 (35)
**Health insurance, n (%)**	12,410 (64)	9813 (71)	26,762 (75)	12,733 (68)	9505 (72)	22,324 (76)
**Last routine checkup, n (%)**						
Within past year	10,880 (57)	8267 (60)	23,541 (65)	12,073 (66)	8665 (68)	20,556 (72)
> 1 year but ≤ 2 years ago	2969 (19)	2195 (18)	5498 (17)	2249 (15)	1530 (14)	3556 (14)
≥ 3 or more years or never	3800 (24)	2691 (22)	6305 (19)	2339 (19)	1819 (18)	3505 (15)
**Didn’t seek care due to cost**^c^**, n (%)**	4210 (25)	2694 (21)	6550 (18)	3943 (27)	2608 (24)	5104 (20)

^a^States that enacted immigrant-inclusive license policies, expanded Medicaid, and were included in the analysis: California, Colorado, Connecticut, District of Columbia, Illinois, Maryland, Nevada, and Vermont. Participants living in Hawaii, New Mexico, Utah, and Washington were excluded because their inclusive policies were enacted prior to 2011; Participants living in Delaware were excluded due to small sample size.

^b^States that did not enact immigrant-inclusive policies but expanded Medicaid: Arizona, Arkansas, Iowa, Kentucky, Massachusetts, Michigan, New Hampshire, New Jersey, New York, North Dakota, Ohio, Rhode Island, and West Virginia

^c^Adults who reported there was a time in the past 12 months when they needed to see a doctor but could not because of cost

### Perceived poor physical health days per month

**Fig. 2:**
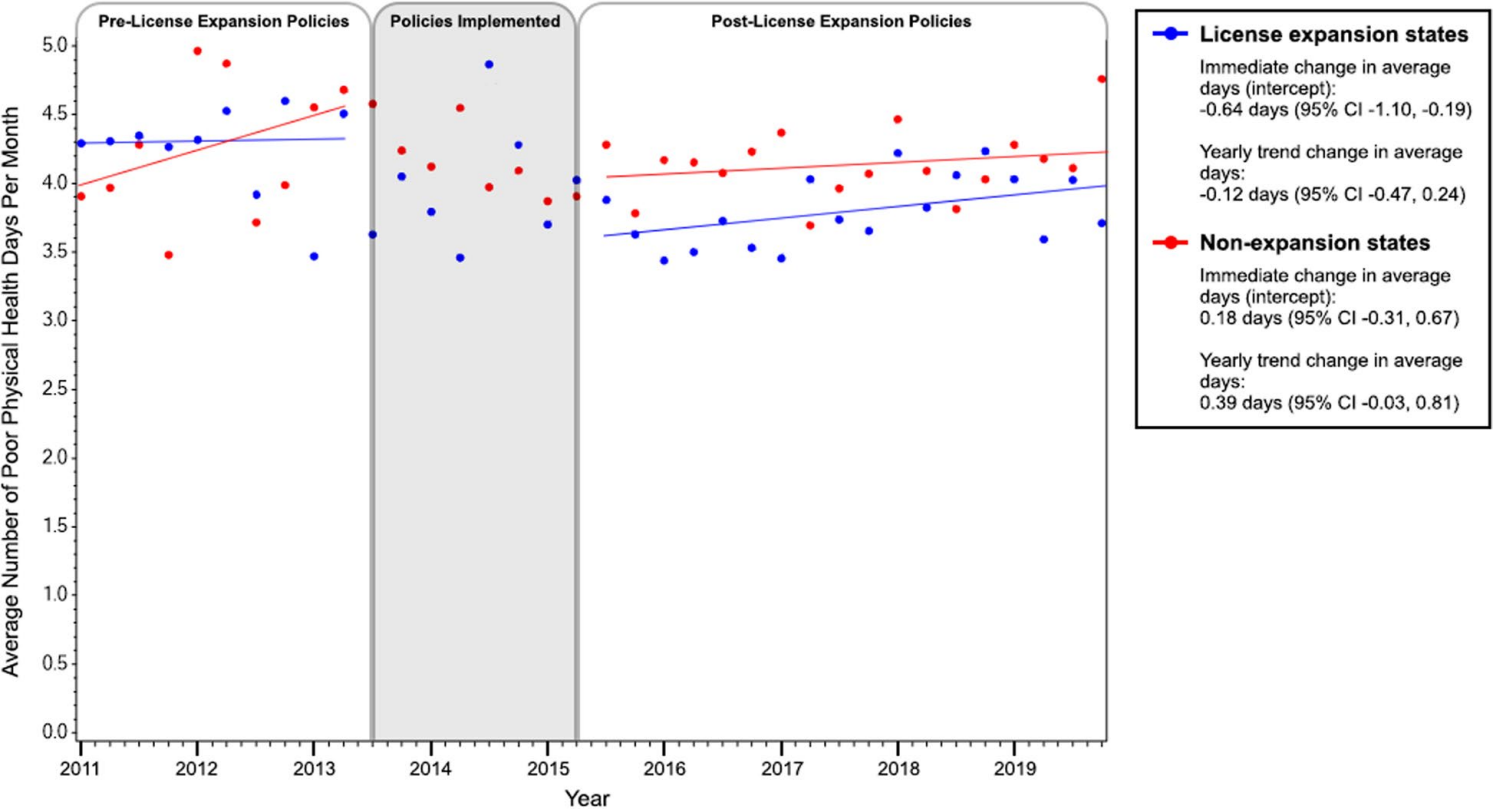
Trends in average number of poor physical health days, before and after the implementation of immigrant-inclusive license policies, stratified by states that did and did not implement these policies

Among Latino adults living in the intervention states, the average number of perceived poor physical health days per month was 4.30 (standard deviation (SD) = 0.09) during the pre-intervention period (January 2011 – June 2013), decreasing to 3.80 (SD = 0.06) in the post-intervention period (July 2015 – December 2019). The average number of perceived poor physical health days per month remained relatively consistent in the pre-implementation period (yearly change in number of perceived poor physical health days per month = 0.39, 95% CI = -0.03 to 0.81). After all immigrant-inclusive license policies were enacted (July 2015), the average number of perceived poor physical health days significantly decreased by over half a day each month (immediate change = -0.64, 95% CI = -1.10 to -0.19), Fig. 2. After this initial drop, the average number of perceived poor physical health days per month remained consistent during the post-implementation period (yearly change in number of poor physical health days per month = -0.12, 95% CI = -0.47 to 0.24).

Conversely, among Latino adults living in control states, the average number of perceived poor physical health days per month was 4.28 (SD = 0.13) during the pre-intervention period, decreasing slightly to 4.13 (SD = 0.08) in the post-intervention period. Overall, the average number of perceived poor physical health days per month remained relatively consistent in the pre-implementation and post-implementation period (immediate change = 0.18, 95% CI = -0.31 to 0.66; yearly change in number of perceived poor physical health days per month = 0.39, 95% CI = -0.03 to 0.81).

When perceived poor physical health days was dichotomized (≥ 1 day vs. none), the proportion of Latino adults reporting ≥ 1 poor physical health day each month decreased from 41% during pre-intervention to 34% post-intervention among those living in intervention states, Table 2. Among those living in control states, the proportion reporting ≥ 1 poor physical health day each month slightly decreased from 39% during pre-intervention to 37% post-intervention. After immigrant-inclusive policies were implemented, there was a 11% decrease in the odds of reporting ≥ 1 poor physical al health days in those states (OR = 0.89, 95% CI = 0.80–1.00). This decrease was not observed among control states (OR = 0.97, 95% CI = 0.85–1.10), Table 2.

**Table 2:** Prevalence of any perceived poor physical or mental health days, stratified by intervention status and study time period, weighted to be nationally representative, BRFSS 2011–2019

	Enacted immigrant-inclusive license policies^a^		Did not enact inclusive immigrant-inclusive policies^b^	
	% (95% CI)	OR (95% CI)^c^	% (95% CI)	OR (95% CI)^c^
**Perceived poor physical health**				
Pre-intervention	40.9 (39.9–42.1)	1.0 (ref)	38.5 (37.1–39.9)	1.0 (ref)
Post-intervention	33.9 (33.2–34.7)	0.89 (0.80, 1.00)	36.7 (35.8–37.5)	0.97 (0.85, 1.10)
**Perceived poor mental health**				
Pre-intervention	40.1 (38.9–41.2)	1.0 (ref)	38.2 (36.8–39.7)	1.0 (ref)
Post-intervention	32.9 (32.2–33.6)	0.84 (0.74, 0.94)	35.5 (34.7–36.3)	0.91 (0.77, 1.09)

^a^States that enacted immigrant-inclusive license policies, expanded Medicaid, and were included in the analysis: California, Colorado, Connecticut, District of Columbia, Illinois, Maryland, Nevada, and Vermont. Participants living in Hawaii, New Mexico, Utah, and Washington were excluded because their inclusive policies were enacted prior to 2011; Participants living in Delaware were excluded due to small sample size

^b^States that did not enact immigrant-inclusive policies but expanded Medicaid: Arizona, Arkansas, Iowa, Kentucky, Massachusetts, Michigan, New Hampshire, New Jersey, New York, North Dakota, Ohio, Rhode Island, and West Virginia

^c^Comparison of the quarterly prevalence of having at least one perceived poor physical (or mental) health day per month before and after a statewide immigrant-inclusive license policy was enacted; among control states, prevalence of perceived poor physical and mental health were compared across the same time periods

When analyses were restricted to Latino adults with at least one perceived poor physical health day each month, a similar immediate reduction in poor physical health days was seen in intervention states, although confidence intervals were wide (immediate change = -0.67, 95% CI = -1.46 to 0.12). Among Latino adults with at least one perceived poor physical health day each month living in control states, no meaningful immediate change in the number of poor physical health days were seen when we compared the same time periods (immediate change = 0.35, 95% CI = -0.49 to 1.19).

### Perceived poor mental health days per month

**Fig. 3:**
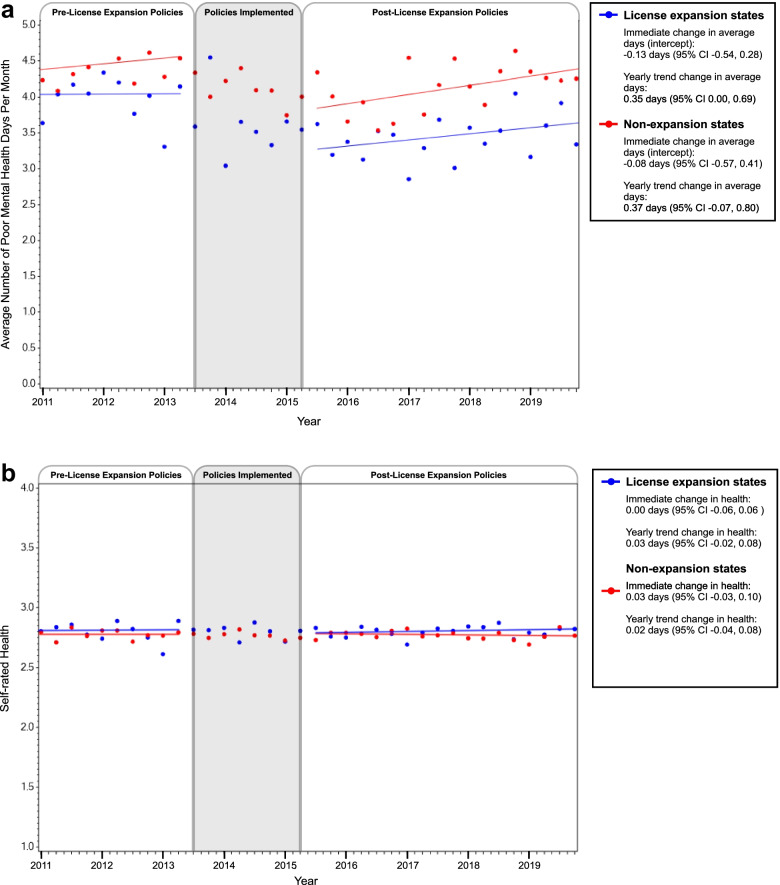
Trends in (**A**) average number of poor mental health days and (**B**) average self-rated general health, before and after the implementation of immigrant-inclusive license policies, stratified by states that did and did not implement

Among those living in intervention states, the average number of perceived poor mental health days per month was 4.03 (SD = 0.09) during the pre-intervention period, decreasing to 3.45 (SD = 0.06) during the post-intervention period. No meaningful changes in the average number of perceived poor mental health days per month was seen after the implementation of immigrant-inclusive license policies (immediate change = -0.13, 95% CI = -0.54 to 0.28; yearly change in slope = 0.08, 95% CI = 0.00 to 0.17), Fig. 3A. Similar results were seen among Latino adults living in control states; the average number of perceived poor mental health days per month was 4.47 (SD = 0.13) during the pre-intervention period, decreasing to 4.11 (SD = 0.07) in the post-intervention period, and no changes in the average number of perceived poor mental health days per month were seen in the post-intervention period (immediate change = -0.08, 95% CI = -0.57 to 0.41; yearly change in slope = 0.13, 95% CI = 0.01 to 0.24), Fig. 3A.

When perceived poor mental health days per month was dichotomized (≥ 1 vs. none), the proportion of Latino adults reporting ≥ 1 poor mental health day each month decreased from 40% during pre-intervention to 33% post-intervention among those living in intervention states, a roughly 17% decrease in the number of Latinos who experienced poor mental health days after immigrant-inclusive license policies were implemented (OR = 0.84, 95% CI = 0.74–0.94), Table 2. Among those in control states, the proportion reporting ≥ 1 poor mental health day each month was 38% during the pre-intervention time period and 35% in the post-intervention time period (OR = 0.91, 95% CI = 0.77–1.09).

When we restricted to those with at least one perceived poor mental health day per month, no changes over time were seen in either those living in intervention states (immediate change = 0.27, 95% CI = -0.45 to 0.98) or control states (immediate change = -0.14, 95% CI = -0.98 to 0.69).

### Perceived general health

Overall, the average perceived general health score was 2.81 (SD = 0.01) among Latinos living in intervention states and 2.77 (SD = 0.01) among those living in control states. No changes in perceived general health were seen over time among those living in either the intervention or control states, Fig. 3B. Also, no change was seen when perceived general health was dichotomized (poor/fair versus good/very good/excellent; case states: OR = 0.94, 95% CI = 0.84 to 1.07; control states: OR = 1.10, 95% CI = 0.96 to 1.26).

## Discussion

In this national analysis of Latino adults, we found that those living in states where immigrant-inclusive license policies were enacted between 2013–2015 (as well as Medicaid expansion) saw an improvement in perceived physical and mental health, whereas states that did not enact such license policies (but did enact Medicaid expansion) did not see similar improvements during the same time period. Specifically, enacting an immigrant-inclusive license policy was associated with an average of 0.64 fewer poor physical health days per month among Latino adults overall, and a 11% reduction in the proportion of Latino adults having poor physical health days each month. And while the average number of perceived poor mental health days did not change after implementation of immigrant inclusive license policies, a 17% reduction in the proportion of Latino adults experiencing poor mental health days was observed. License expansion did not appear to impact perceived general health.

State-level immigrant policies such as limiting access to driver’s licenses (and state identification cards) affect health and well-being at the institutional and individual levels [7]. Lack of personal identification has been identified as a barrier to accessing social services for marginalized populations [20]. Exclusionary immigrant policies, such as the 2010 Arizona Senate Bill 1070 immigration law, have resulted in decreases in the use of preventative healthcare services and public assistance [21]. Access to a driver’s license affects physical and social mobility, employment, mental health, and access to healthcare services and social institutions (schools and social programs) for undocumented Latinos, legal residents, and U.S. citizens [7]. Social program eligibility criteria and policies can also influence social assistance participation. For instance, application forms for many social services require a form of identification [10]. Undocumented immigrants may not have U.S. identification documentation or may fear using their home country identification; thus, the ability to obtain a driver’s license can provide the needed reassurance to drive an automobile and utilize public services.

Possession of a driver’s license is associated with increased vehicle ownership and employment rates by facilitating job accessibility among undocumented immigrants [22]. The ability to expand maximum commuting distance allows for a greater chance of finding employment, working longer hours, and earning higher wages, thus increasing financial stability which positively impacts health [23].

Despite Medicaid expansion, we did not see any change in perceived mental or physical health among Latinos living in states that did not have immigrant-inclusive license policies. This may be explained partly by the “chilling effect” that discourages undocumented immigrants from using public services and being in public places [6, 10]. This “chilling effect” is exacerbated by policies like Secure Communities that require state and local law enforcement to partner with federal immigration authorities [10]. Such policies can increase fear and stress and can have negative health impacts [6]. Potentially detrimental health effects of not having a license, or vice-versa, the salutary effects of having a license, could extend to Latino U.S. citizens and legal residents living with mixed-status families or communities [12]. The stress and fear associated with family members’ legal status has been shown to provoke depression and anxiety among Latino U.S. citizens and has been associated with more cardiovascular risk factors [24, 25]. As has been pointed out previously, racial profiling, e.g., racial discrimination during traffic stops including searches prompted by the driver’s race, is a public health and health equity issue and impacts all Latinos, irrespective of their legal status [14, 15]. Thus, anti-immigrant policies harm the health of immigrant groups living in the U.S. and Latino communities and exacerbate racial health disparities among citizens and non-citizens alike [11].

This study has several strengths and limitations. First, we conducted a national analysis using a quasi-experimental design and a negative control group, which allows us to estimate the effect of immigrant-inclusive license policies on Latino health. While we restricted our intervention and control groups to states which expanded Medicaid in 2014, it is still possible that other policies or external influences, e.g., other health or immigration surveillance policies or programs that differentially affected the implementation and control states could be causing the observed effect. Additionally, these results may not generalize to states that have not expanded Medicaid under the Affordable Care Act. Moreover, because immigration status is not captured in BRFSS, we were unable to differentiate the effects of these policies among undocumented, legal resident, and U.S. citizen Latinos living in the U.S. We were also only able to investigate perceived poor mental and physical health days, which are based on individual perceptions using single-item measures and not validated scales or diagnoses. Finally, although the BRFSS is a nationally representative longitudinal survey that can assess trends and changes over time, individual participants are not followed longitudinally; therefore, we are unable to assess individual-level changes.

## Conclusions

In this multi-state analysis assessing the impact of immigrant-inclusive license policies (i.e., state laws that allow for the issuing of driver’s licenses regardless of immigration status), we found that enacting immigrant-inclusive license policies (2013–2015) decreased the number of perceived poor physical health days per month, the proportion of adults experiencing poor physical health, and the proportion of adults experiencing poor mental health among Latinos. This decrease was not observed among states that did not have inclusive policies, even though they had expanded Medicaid through the Affordable Care Act in 2014. Immigrant-inclusive driver’s license policies deserve further assessment regarding their potential to reduce health disparities via increasing access to employment opportunities, healthcare and social services that might enhance health, and decreasing racial profiling, fears of deportation, and stress that can harm health. Because anti-immigrant policies can harm Latino communities regardless of immigration status and further widen health inequities, implementing state policies that do not restrict access to driver licenses based on immigrant status documentation could help address upstream drivers of such inequities.

## Data Availability

The datasets generated during and/or analyzed during the current study are available in the Behavior Risk Factor Surveillance System repository, [https://www.cdc.gov/brfss/].

## Abbreviations

U.S.: United States
BRFSS: Behavioral Risk Factor Surveillance System
SD: Standard deviation
CI: Confidence interval
OR: Odds ratio
